# Topographic patterns of retinal edema in eyes with branch retinal vein occlusion and their association with macular edema recurrence

**DOI:** 10.1038/s41598-021-02726-w

**Published:** 2021-12-01

**Authors:** Hae Min Park, Young Hwan Kim, Byung Ro Lee, Seong Joon Ahn

**Affiliations:** Department of Ophthalmology, Hanyang University Hospital, Hanyang University College of Medicine, 222 Wangsipli-ro, Seongdong-gu, Seoul, 04763 Republic of Korea

**Keywords:** Retinal diseases, Predictive markers

## Abstract

In this study, we evaluated the topographic pattern of retinal edema in eyes with macular edema (ME) secondary to branch retinal vein occlusion (BRVO) using a widefield retinal thickness map of optical coherence tomography and its association with ME recurrence. In 87 eyes with ME secondary to BRVO who were treated with anti-vascular endothelial growth factor (VEGF) injections and followed up for ≥ 1 years, 12 × 9 mm macular volume scans of swept-source optical coherence tomography (DRI-OCT Triton; Topcon Inc, Japan) were performed and retinal thickness maps were automatically generated at baseline and follow-up visits. Topographic patterns of retinal edema on the maps at baseline and 1 month after the first anti-vascular endothelial growth factor (VEGF) treatment were classified as extramacular (outside the ETDRS grid), macular (within the grid), and combined pattern and correlated with ME recurrences. Seventy-five of 87 (86.2%) eyes with BRVO ME showed combined edema at baseline. There were 4 topographic patterns of edema at 1 month following anti-VEGF injection as follows: no residual edema, extramacular only, macular only, and combined edema. In contrast to the baseline pattern, the pattern of retinal edema 1 month following anti-VEGF therapy showed significant association with 6-month recurrence, number of ME recurrences during a 1-year period, and time to first recurrence. (all *P* < 0.05) An automatically generated widefield retinal thickness map could be used to effectively visualize the topographic patterns of retinal edema in eyes with BRVO. The map can be used as a valuable tool for detection of retinal edema on widefield retinal areas and prediction of ME recurrence in eyes with BRVO.

## Introduction

Retinal vein occlusion (RVO), vascular occlusions of the central retinal vein or its branch, is the second most common retinal vascular disease after diabetic retinopathy^1,2^. Previous studies have estimated the prevalence of RVO to be 0.3–1.6%, and branch retinal vein occlusion (BRVO) is the more common of the two main types of RVO^3–5^. It is associated with several complications, including macular edema (ME), neovascular glaucoma, and vitreous hemorrhage, which can lead to vision loss in patients^1^.

Macular edema is a common complication of BRVO and the most common cause of visual decline in patients with BRVO^1,2^. The pathophysiology of ME is characterized by increased vascular permeability due to upregulation of vascular endothelial growth factor (VEGF) caused by vascular occlusion^6,7^. Accordingly, anti-VEGF agents are widely used for the treatment of ME secondary to BRVO, and several trials have shown successful results in terms of anatomic and visual outcomes. However, recurrence of ME is common in patients with BRVO, and regular monitoring and repeated injections of anti-VEGF agents are necessary.

Prediction of ME recurrence is clinically important for long-term outcomes in patients with ME due to BRVO. Several predictive factors, including disorganization of the retinal inner layer and changes in the macular capillary network, have been reported^8,9^. A widefield (12 × 9 mm) retinal thickness map can be created using 3D volume scan of commercially available swept-source (SS) OCT machines (DRI-OCT Triton; Topcon Inc., Tokyo, Japan); in the map, retinal thickness can be visualized as color codes on a wide area of interest. The area scanned by SS-OCT covers the optic disc, macula, and the major vascular arcade. Using this map, we aimed to investigate the topographic pattern of changes in retinal thickness in eyes with retinal edema associated with BRVO. In particular, we investigated the topographic patterns of retinal edema at baseline and 1 month after anti-VEGF therapy and correlated them with ME recurrences over a follow-up period. Our study aimed to address the clinical implication of this novel imaging technique for evaluating ME secondary to BRVO.

## Results

### Clinical characteristics

The demographic data and clinical characteristics of the patients, including 35 men and 52 women, enrolled in this study are summarized in Table 1. The mean age of the patients was 63.6 ± 10.5 years. The eyes with ME associated with BRVO included 49 and 38 eyes with superior and inferior RVO, respectively, and 37 and 50 eyes with macular and major BRVO, respectively. The mean follow-up period of the patients was 27.9 ± 21.0 months. The mean central macular thickness (CMT) and best-corrected visual acuity (BCVA) was 454.0 ± 142.5 μm and 0.53 ± 0.31 logMAR, respectively, at baseline. After treatment, CMT decreased to 242.6 ± 42.1 μm and BCVA was improved to 0.34 ± 0.26 logMAR at the 1-year visit. Supplementary Fig. S1 shows the relationship between the CMT changes and BCVA improvement. In 71 of 87 (81.6%) eyes, macular edema recurred during the follow-up period, whereas the others (n = 16) showed no recurrence during the study period.

**Table 1:** Baseline characteristics and treatment details of the included patients with macular edema secondary to branch retinal vein occlusion (n = 87).

Characteristics	Number (% or range)
Age, years	63.6 ± 10.5 (39–89)
Sex, female (%)	52 (59.8%)
Location of vascular occlusion, Superotemporal:inferotemporal	50 (55.6%):37 (42.2%)
Subtype of branch retinal vein occlusion, Macular:major (%)	37 (42.5%):50 (57.5%)
Baseline BCVA, logMAR	0.53 ± 0.31 (0.05–1.70)
Follow-up period, months	27.9 ± 21.0 (12–71)
Central macular thickness, μm	454.0 ± 142.5 (300–912)
**Baseline topographic patterns**	
Macular only (%)	12 (13.8%)
Combined edema (macular dominant, %)	28 (32.2%)
Combined edema (extramacular dominant, %)	47 (54.0%)
Anti-VEGF agents Bevacizumab:ranibizumab (%)	69:18 (79.3%:20.7%)
Number of injections over a 1-year period*	3.2 ± 1.6 (1–9)

Continuous values are denoted as mean ± standard deviation.

*BCVA* best-corrected visual acuity, *VEGF* vascular endothelial growth factor, *logMAR* logarithm of the minimum angle of resolution.

*Including the first injection at baseline.

### Baseline pattern of macular edema

**Figure 1:**
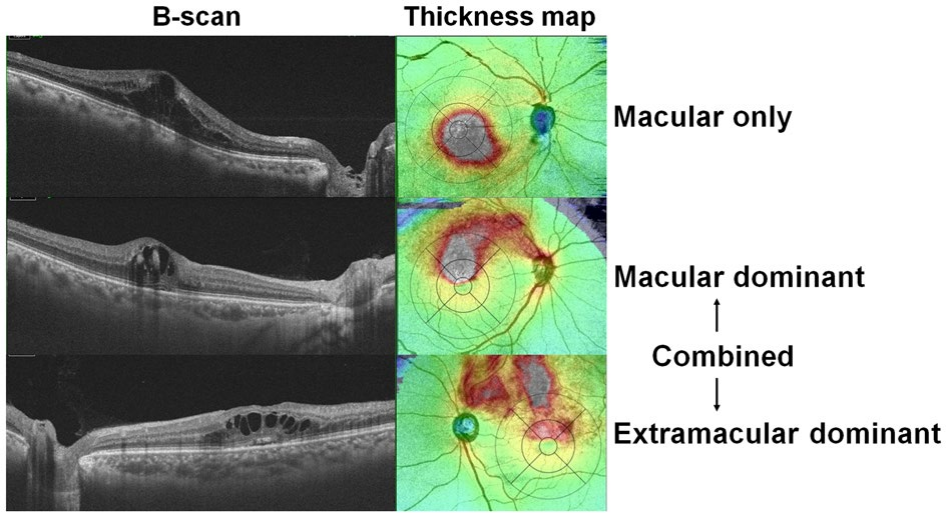
Pattern of retinal edema at baseline in eyes with macular edema secondary to branch retinal vein occlusion (BRVO). All horizontal B-scans across the fovea shows cystoid macular edema. However, the extent and topographic features of retinal edema vary from edema exclusively in the macula (**A**) and combined extramacular and macular edema (**B**, **C**). Depending on the dominant area of edema (white or red areas), eyes with combined edema were separated into macular dominant (**B**) and extramacular dominant edema (**C**).

Figure 1 shows representative widefield retinal thickness map images of eyes with ME secondary to BRVO at baseline, generated by a commercially available OCT image viewer software (IMAGEnet 6, Topcon, Inc., Tokyo, Japan; Supplementary Fig. S2). Among those with ME, retinal edema confined within the ETDRS grid was seen only in 12 eyes (13.8%); most of those with ME (75, 86.2%) had combined macular and extramacular edema. Those with combined edema were further divided; the dominant area of edema, as determined by the comparison of the edematous areas within and outside the ETDRS grid, was observed within (macular dominant) and outside (extramacular dominant) the macula in 28 (32.2%) and 47 (54.0%) eyes, respectively.

Among the three topographic groups, however, there were no significant differences in the baseline characteristics, such as CMT (*P* = 0.502 by analysis of variance (ANOVA)) and baseline BCVA (*P* = 0.784), as presented in Supplementary Table S1 Baseline topographic patterns of retinal edema were significantly associated with subtype of BRVO (*P* < 0.001). However, the number of recurrences during the 1-year follow-up period and the frequency of eyes with 6-month recurrence were not statistically different between the groups (*P* = 0.673 and 0.864, respectively; Supplementary Table S1). Additionally, there were no significant differences in the number of recurrences during the 1-year follow-up period (*P* = 0.413) and number of eyes with recurrence within 6 months (*P* = 0.733) between the patients with ME only and those with combined edema.

### Pattern of retinal edema following anti-vascular endothelial growth factor therapy

**Figure 2:**
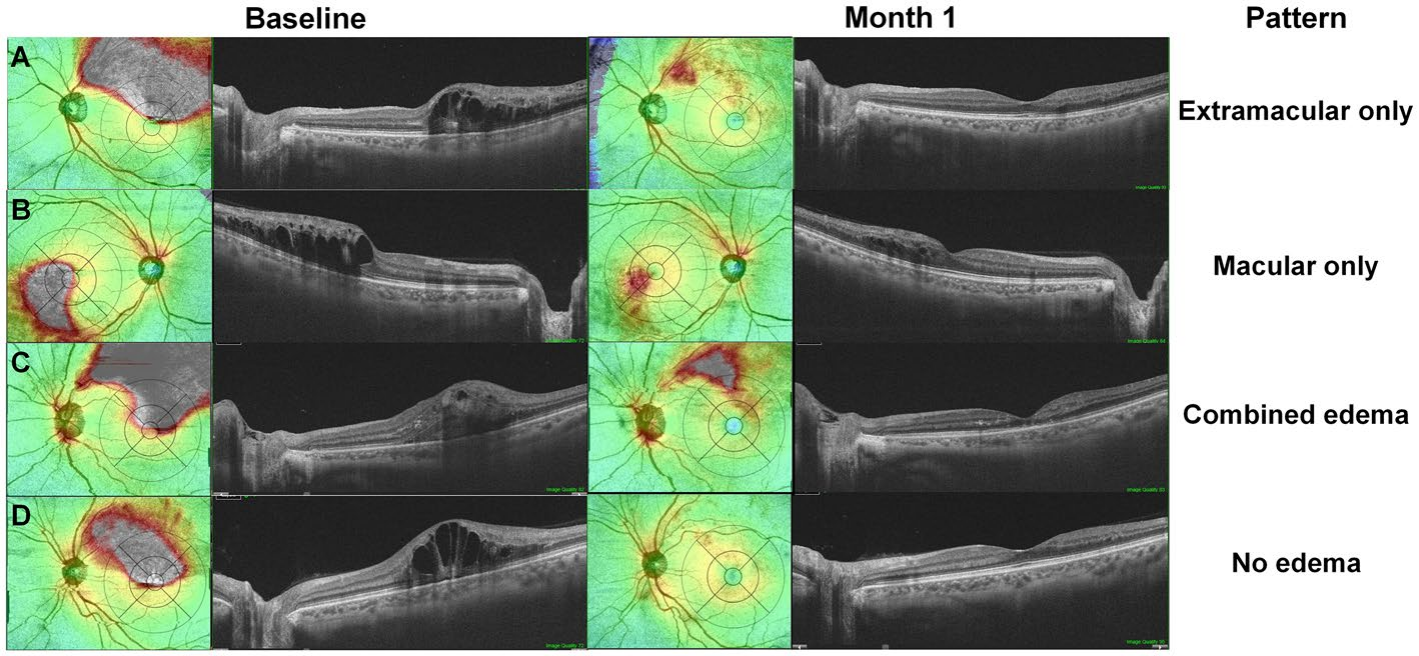
Pattern of retinal edema 1 month following anti-vascular endothelial growth factor (VEGF) therapy for macular edema (ME) due to branch retinal vein occlusion (BRVO). Four patterns, (extramacular only [**A**], macular only [**B**], combined edema [**C**], and no edema [**D**]) are noted from the widefield retinal thickness maps. The areas of residual edema are mostly wedge-shaped and located between the fovea and site of vascular occlusion.

Four topographic patterns of retinal edema were observed 1 month after the initial anti-VEGF therapy, as exemplified in Fig. 2. The patients with ME due to BRVO showed no residual edema (Fig. 2A), macular residual edema (Fig. 2B), extramacular (Fig. 2C) residual edema, or combined residual edema (Fig. 2D) in 9 (10.3%), 30 (34.5%), 6 (6.9%), and 42 (48.3%) eyes, respectively (Table 2). The treatment response to anti-VEGF therapy varied, although there were no significant differences in the baseline characteristics including demographic and clinical characteristics and anti-VEGF agents used, among the four groups (Supplementary Table S2). However, the edema patterns at 1 month after anti-VEGF therapy were significantly associated with the subtype of BRVO (*P* < 0.001).

**Table 2:** Parameters of macular edema recurrence in groups of patients separated according to retinal edema pattern at 1 month after initial anti-vascular endothelial growth factor (VEGF) therapy (n = 87).

Parameters	Extramacular only (n = 6)	Macular only (n = 30)	Combined edema (n = 42)	No residual edema (n = 9)	*P* value
6-month recurrence, No./total (%)	3 (50%)	18 (60%)	40 (95.2%)	2 (22.2%)	< 0.001
Number of recurrences in 1 year	1.5 ± 1.5	2.0 ± 1.5	2.9 ± 1.5	0.7 ± 0.7	< 0.001
Median time to first recurrence, months	4.5	4.3	3.1	8.5	< 0.001

Continuous values are denoted as mean ± standard deviation.

**Figure 3:**
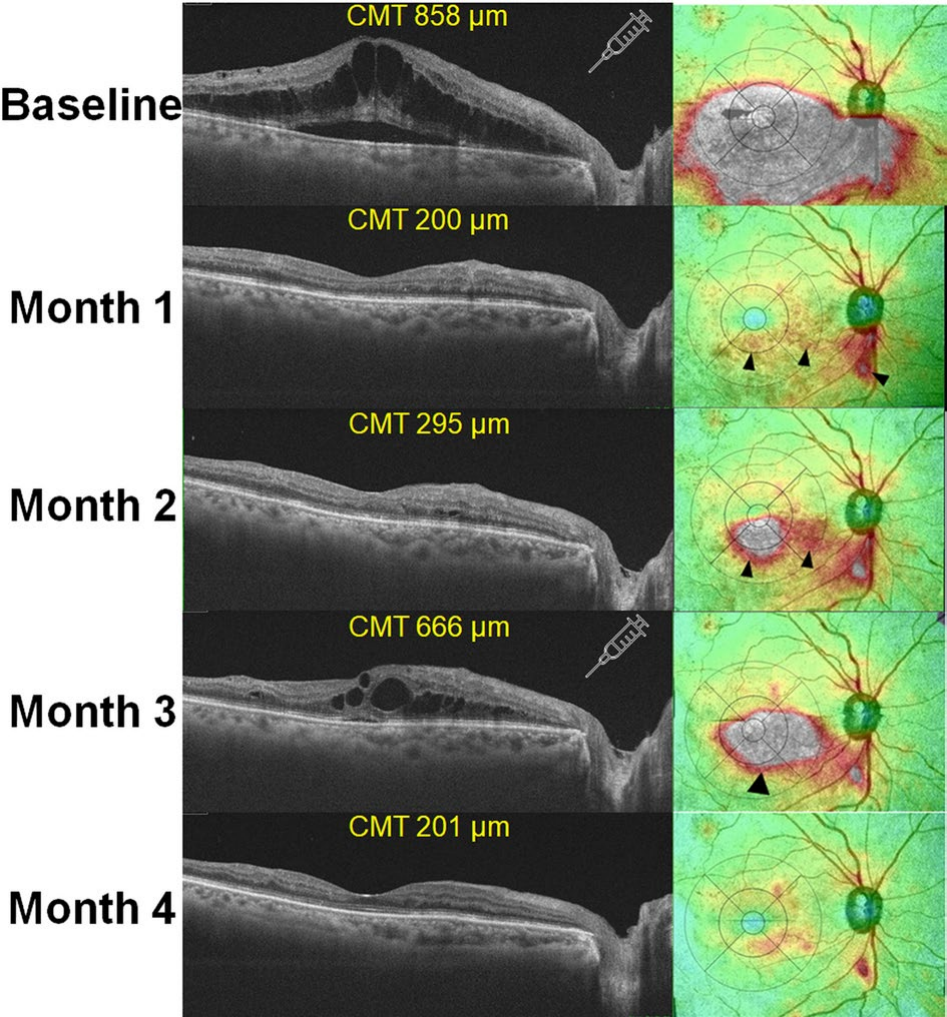
Optical coherence tomography B-scans and retinal thickness maps demonstrating the evolution of macular edema (ME) recurrence over time. At 1 month following initial anti-vascular endothelial growth factor (VEGF) therapy, residual edema remained in the peripapillary and para/perifoveal areas (arrowheads). At month 2, the residual edema in the macular areas was aggravated and expanded. Subsequently, the edematous areas further enlarged and merged into a larger area, leading to recurrence of ME (central macular thickness [CMT] > 300 μm). The syringe icons indicate the timing of anti-VEGF therapy for ME.

Figure 3 shows the clinical course of an eye with ME secondary to BRVO, demonstrating the progression from resolved to recurrent ME on a widefield retinal thickness map. This representative case demonstrates the typical course of ME recurrence, as 59 of 71 (83.1%) eyes with recurrence showed a similar pattern for their first recurrent ME, which occurred due to progression from residual macular and/or extramacular edema. However, as demonstrated in Supplementary Fig. S3, ME reappeared independently (not contiguously) from the residual edema (n = 9) or without any predisposing findings (n = 3).

### Association between macular edema recurrence and post-treatment retinal edema

Overall, the mean number of ME recurrences during the 1-year period was 2.2 ± 1.6. In contrast to the baseline pattern, the pattern of retinal edema 1 month after anti-VEGF therapy was significantly associated with the number of ME recurrences during the 1-year period. As shown in Table 2, the mean number of recurrences during the period was significantly different among the four groups separated according to the pattern of retinal edema (*P* < 0.001). The median time from initial anti-VEGF therapy to first recurrence was 4.5, 4.3, 3.1, and 8.5 months in the extramacular only, macular only, combined edema, and no residual edema groups, respectively. Patients with residual edema in the macular and extramacular areas had the highest number of ME recurrences during the 1-year period (2.9), whereas those without residual edema in the macular or extramacular areas had an average of 0.7 recurrences during the 1-year period.

Furthermore, recurrence in the first 6 months following anti-VEGF therapy was significantly associated with the pattern, as shown in Table 2. For instance, 95.2% of patients with residual edema in the macular and extramacular areas had recurrence during the 6-month period, whereas 50% and 22.2% of patients with residual edema in the extramacular area only and those without residual edema had ME recurrence during the 6-months period, respectively.

**Figure 4:**
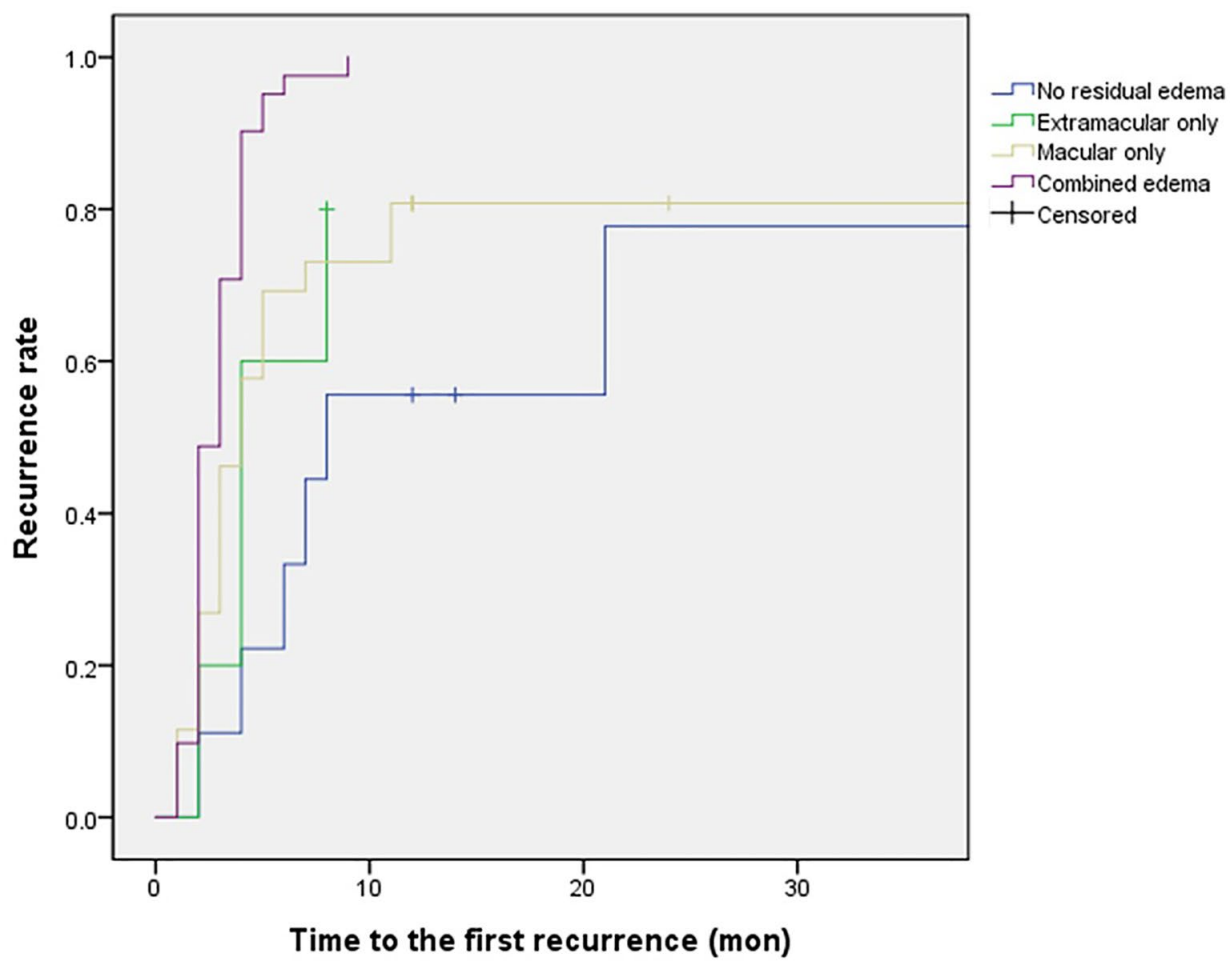
Kaplan–Meier plot of time to the first recurrence in four groups of patients separated based on pattern of retinal edema 1 month following anti-vascular endothelial growth factor (VEGF) therapy for macular edema.

Figure 4 demonstrates the Kaplan–Meier plots of time to recurrence for patients separated according to the pattern of retinal edema 1 month following anti-VEGF therapy. The Kaplan–Meier plot shows that the estimated curve (recurrence) for the group with combined residual edema in the macular and extramacular areas was greater than those for the other groups. In addition, the estimated curves for the groups with edema in either extramacular or macular areas lay above that for those without residual edema. These differences among the groups were statistically significant (*P* < 0.001 by log-rank test).

The results of fitting a proportional hazards model, including both baseline and post-treatment retinal edema patterns, are shown in Table 3. In contrast to the baseline pattern, the post-treatment (1-month) pattern of retinal edema had a statistically significant effect on recurrence, with an estimated hazard ratio for combined macular and extramacular residual edema of 4.58 (95% CI 1.72–12.2; *P* = 0.002) after adjusting for other covariates. However, the agent used for anti-VEGF therapy (bevacizumab vs. ranibizumab) showed no significant relationship with the recurrence rate according to the regression analyses (*P* = 0.698).

**Table 3:** Findings of a fitted proportional hazard model for macular edema recurrence.

Variables	Coefficient	Standard error	HR (95% CI)	*P* value
Age	0.016	0.014	1.02 (0.99–1.04)	0.227
Sex (female to male)	0.259	0.275	1.30 (0.76–2.22)	0.346
Initial central macular thickness	0.001	0.001	1.00 (1.00–1.00)	0.458
Anti-VEGF agent (ranibizumab to bevacizumab)	0.124	0.319	1.13 (0.61–2.11)	0.698
**Pattern of edema**				
At baseline (combined edema to macular edema only)	0.105	0.293	1.11 (0.63–1.97)	0.721
At 1 month following anti-VEGF therapy (combined edema to no residual edema)	1.522	0.501	4.58 (1.72–12.2)	**0.002**

*HR* hazard ratio, *CI* confidence interval, *VEGF* vascular endothelial growth factor.

## Discussion

This study demonstrated different topographic patterns of retinal edema and their association with ME recurrence in patients with BRVO. Residual edema following initial anti-VEGF therapy in the macular and extramacular areas, which could be identified using a widefield retinal thickness map, was significantly associated with recurrence of ME. Accordingly, our results highlight the clinical usefulness of topographic analyses of retinal edema for the prediction of ME recurrence in patients with BRVO.

Although ME is effectively and safely managed with anti-VEGF therapy, recurrence remains a major issue in the management of ME secondary to BRVO**.** According to previous literature, the mean number of anti-VEGF injections based the on pro-re-nata strategy widely ranged from 1.7 in the first year of treatment to 3.2 during a 6-month period^10–12^. Although most patients respond to anti-VEGF therapy, 66–87% still require an additional injection for recurrent ME^13^. Accordingly, the monitoring and prediction of ME recurrence is important for long-term management of BRVO^14^.

OCT is one of the most important imaging modalities for the evaluation and management of BRVO. It provides qualitative and quantitative assessment of the macula, and as a result, is used extensively for the detection of BRVO-associated ME and monitoring of treatment response to anti-VEGF therapy. Several studies have investigated the OCT features related to ME recurrence. Macular cystic changes and non-perfusion areas on superficial and deep capillary plexuses have been reported to be associated with ME recurrence in eyes with BRVO^15,16^. However, these parameters only focus on the morphologic features of OCT or OCT angiography, which require careful investigation and interpretation of the cross-sectional retinal images by experts or complex analyses of OCTA images. On the contrary, a color-coded retinal thickness map does not require subjective interpretation based on morphologic characteristics and may minimize observer-to-observer variability. Moreover, a color-coded map based on retinal thickness measurement is automatically generated using image viewer software without the need for additional time or effort.

Our results highlight that retinal edema is extensively located over the extramacular areas in eyes with ME secondary to BRVO, and residual edema in macular or extra-macular areas may progress to clinically evident ME recurrence. Previous studies have suggested that accumulated fluid diffuses from the area of leakage to the macula in eyes with BRVO, which explains the development of ME from distant BRVO^17–19^. Residual retinal edema around the area of vascular occlusion following the treatment may further contribute to the recurrence of ME by fluid accumulation from the extramacular areas^20,21^. From the association between the combined edema and ME recurrence, our results justify the evaluation of extramacular areas to identify the potential source of ME recurrence following treatment.

However, it was not possible to observe the pattern of edema on the wide retina, particularly in the area around the site of vascular occlusion, on conventional OCT with a 6-mm-width coverage. Therefore, the association between the topographic pattern of edema over the wide retinal areas, including both the macula and vascular occlusion sites, and ME recurrence could not be evaluated. However, we used a 12 × 9 mm volume scan of a commercially available SS-OCT software, which generated widefield retinal thickness maps covering the peripapillary, macular, and extramacular areas. This map highlighted most of the edematous areas in eyes with BRVO and the severity of edema using color coding. The response to anti-VEGF therapy could be reclassified based on the topographic pattern of edema, as determined using an ETDRS grid within the map, and ME recurrence could be predicted from the topographic pattern. Furthermore, as demonstrated in Fig. 3, the temporal change in the topography of retinal thickness provided valuable insights into the evolution of ME recurrence in eyes with BRVO and the role of residual edema following anti-VEGF therapy.

The areas of residual edema were mostly wedge-shaped and were commonly located between the fovea and site of vascular occlusion, as depicted in Fig. 2. Increased intraluminal venous pressure following acute vein occlusion may lead to increased hydrostatic pressure in the affected retinal capillaries of eyes with acute BRVO^7^. Subsequently, cystoid spaces may be formed with serous fluid leakage from the blood into the retina and its accumulation^7^. Although anti-VEGF therapy reduces the leakage and edema by decreasing vascular permeability, hemodynamic instability might not be completely resolved, potentially leading to residual edema in the area. Furthermore, capillary dropout in the affected retina of eyes with BRVO is reported to cause upregulation of angiogenic cytokines such as VEGF^7,20,21^. Overexpression of VEGF due to hypoxia associated with capillary nonperfusion may also explain the localized residual edema following anti-VEGF therapy. Because of the short-term efficacy of bevacizumab or ranibizumab, VEGF expression may increase again and induce localized edema 1 month following the therapy. Multiple studies have shown an association between capillary nonperfusion and an increased rate of ME recurrence in RVO and other retinal vascular disorders after anti-VEGF treatment^8,10^. Although we were unable to validate the association between residual edema and capillary nonperfusion, residual edema, as the initial sites of retinal changes induced by VEGF expression due to capillary nonperfusion, might mediate the pathogenic association between capillary nonperfusion and ME recurrence.

However, several limitations of our study need to be carefully considered when interpreting our data. First, the heterogeneity in anti-VEGF agents may bias our results, particularly the outcome measures of recurrence. However, several recently published studies, such as the Bevacizumab versus Ranibizumab in Branch Retinal Vein Occlusion (MARVEL) study and the bevacizumab versus ranibizumab in treatment of macular edema from vein occlusion (CRAVE) study, showed comparable therapeutic response for BRVO-related ME with the two drugs^12,22,23^. In our analyses, the difference in anti-VEGF agents was not significantly associated with the pattern of retinal edema at baseline and after initial anti-VEGF therapy nor with the outcomes of ME recurrence. Accordingly, our results further support the comparability of treatment outcomes related to recurrence between bevacizumab and ranibizumab. Second, the retrospective nature of our study and exclusion of patients who received steroid therapy for ME during the 1-year period may be sources of selection bias. These patients were mostly refractory to anti-VEGF therapy; therefore, severe or refractory cases were likely to have been excluded from our study population. Future studies evaluating retinal edema patterns after steroid treatment and comparison with the results obtained from the anti-VEGF treatment group are required to extrapolate our results to the general population with BRVO. Our data obtained using OCT equipment and software from a single vendor should be validated using different OCT devices. As other commercially available devices also offer color-coded retinal thickness maps, our findings should be confirmed using other devices for the maps to be widely applicable across the instruments. Finally, segmentation errors should be carefully examined when interpreting retinal thickness maps. Although precise segmentation between the internal limiting membrane and retinal pigment epithelium was achieved using high-quality SS-OCT images in most of the included patients, pathologic findings such as hemorrhage, cotton-wool patches, and exudate may interfere with proper automated segmentation, which should be manually corrected.

In summary, this study used a widefield retinal thickness map for eyes with BRVO to demonstrate topographic patterns of ME, and edematous eyes could be classified according to the patterns. The pattern 1 month after anti-VEGF therapy showed a significant association with ME recurrence. Considering the ease of image acquisition and interpretation, this map may be a valuable tool to determine potential sources of recurrence, understand the evolution of ME recurrence, and predict future recurrence in patients with BRVO-related ME.

## Methods

### Study participants

This study included 183 consecutive patients with acute BRVO and associated ME on SS-OCT (DRI-OCT Triton; Topcon, Tokyo, Japan) who visited Hanyang University Hospital between March 2015 and June 2020. The diagnosis of BRVO was made by retina specialists on the basis of the typical funduscopic features of BRVO, such as retinal hemorrhage or cotton wool patch limited to areas associated with occluded vessels, and/or angiographic evidence on fluorescein angiography. Patients with coexisting retinal diseases, such as combined retinal vascular diseases (n = 10) and macular diseases other than macular edema (n = 8), that may have affected retinal thickness were excluded. Patients who had previously received treatment, including anti-VEGF therapy and laser treatment (n = 19), and those who received any treatment other than anti-VEGF therapy for ME during a 1-year follow-up period (n = 17) were also excluded. Furthermore, patients with a follow-up period < 12 months (n = 37) and those with poor-quality OCT images (quality score < 60, motion artifacts, or decentration of the macular Early Treatment Diabetic Retinopathy Study (ETDRS) grid that could not be corrected due to severe pathology; n = 5), which prevented accurate retinal thickness analysis, were excluded. Finally, 87 eyes of 87 treatment-naïve patients with acute BRVO and associated ME were included in our analyses. This retrospective study was approved by the Institutional Review Board (IRB) of Hanyang University Hospital and adhered to the tenets of the Declaration of Helsinki^24^. The need for informed consent was waived by the IRB due to the retrospective nature of the study.

### Examinations

All included patients underwent complete ophthalmic examinations at baseline and every follow-up visit. The examinations included BCVA assessment using a Snellen chart, automated refraction (KW-1500; Kowa, Tokyo, Japan), noncontact tonometry (KT-500 automated tonometer; Kowa), slit-lamp examination, and fundus photography with pupil dilation. Snellen visual acuities were converted into logarithm of minimal angle of resolution (logMAR) for statistical analysis. At each visit, SS-OCT was performed using the 3D macular volume scan protocol of the DRI-Triton OCT system. This protocol generated a cube of data through a 12 × 9 mm grid, which included the macula, optic disc, and major vascular arcade. All included patients first received anti-VEGF treatment at the baseline visit, and follow-up examinations were repeated at 1-month intervals over the 1-year follow-up period.

The subtypes of BRVO were categorized based on the site of vascular occlusion seen in the color photographs of fundus in accordance with previous definitions^25,26^. Major BRVO was defined as an occlusion of a temporal arcade vein or branch extending to the peripheral retina beyond the retinal vascular arcades, whereas macular-type BRVO was defined as an occlusion confined between the superior and inferior retinal temporal vascular arcades.

### Assessment of retinal edema using a widefield retinal thickness map

Supplementary Fig. S2 shows the details of a widefield retinal thickness map generated by a commercially available OCT image viewer software (IMAGEnet 6, version 1.24.1.15742; Topcon, Inc., Tokyo, Japan). The segmentation for retinal thickness measurement was automatically performed from the internal limiting membrane to the retinal pigment epithelium using the software provided by the manufacturer. The segmentation lines for all included images were reviewed, and any errors were manually corrected by an independent reviewer (S.J.A.). The position of the ETDRS grid on the macula was assessed, and decentration errors, if any, were manually corrected. Each widefield retinal thickness map consisted of 512 × 256 color-coded (yellow box) pixels within a 12 × 9-mm^2^ area of the posterior pole. The red and white pixels on this map were considered to have abnormality, retinal thickening.

The topographic pattern of retinal edema was classified based on the location of retinal thickening. Depending on the location of the edema with respect to the macular ETDRS grid, retinal edema was classified as either macular (within the ETDRS grid) or extramacular (outside the grid) edema. If retinal edema occurred concurrently in both macular and extramacular areas, it was classified as having a combined pattern. The retinal thickness map images were evaluated independently by two reviewers (H.M.P. and S.J.A.) who were blinded to the clinical information. Any discrepancy between the two reviewers was resolved by consensus through discussion.

### Macular edema treatment

Macular edema was defined as a CMT ≥ 300 μm based on the ETDRS protocol, which involved segmentation of the macula into a central circle, inner ring, and outer ring of 1-, 3-, and 6-mm diameters. The CMT was measured by calculating the average retinal thickness in a circle of 1 mm diameter centered on the fovea, and measuring the distance between the first signal from the vitreoretinal interface and the outer border of the retinal pigment epithelium. Recurrence of ME was defined as the reappearance of ME in eyes with previously resolved ME (CMT < 300 μm).

Macular edema was treated with as-needed intravitreal injections of anti-VEGF agents, bevacizumab (1.25 mg; Avastin, Genentech, San Francisco, CA, USA) or ranibizumab (Lucentis, 0.5 mg; Genentech, Inc., San Francisco, CA, USA). If three consecutive monthly anti-VEGF injections failed to resolve ME, the patient was switched to intravitreal dexamethasone implant or triamcinolone acetonide injections.

### Analyses

Descriptive statistics were used to assess patient demographics and clinical characteristics. The mean number of ME recurrences during the 1-year follow-up period were compared between the groups separated by the pattern of retinal edema on widefield retinal thickness maps at baseline and at 1 month after initial anti-VEGF therapy using analysis of variance (ANOVA) or Kruskal–Wallis test. Kaplan–Meier survival curves were generated to assess the time from initial anti-VEGF therapy to ME recurrence in groups of patients separated according to the pattern of retinal edema. The log rank test was used to compare the survival distributions among the groups. Further, multivariable Cox proportional hazard regression modeling was used to evaluate clinical factors that affected ME recurrence. Continuous variables are presented as means ± standard deviations. For all analyses, *P* values < 0.05, were considered statistically significant. Statistical analyses were performed using SPSS software version 23 (IBM Corp., Armonk, NY, USA).

## Supplementary Information

Supplementary Information.
