# Supplementary material for: Topographic patterns of retinal edema in eyes with branch retinal vein occlusion and their association with macular edema recurrence
Source: Sci Rep. 2021 Dec 1;11:23249. doi: 10.1038/s41598-021-02726-w (PMC8636476; doi:10.1038/s41598-021-02726-w)
Supplement: Supplementary file 1 — Supplementary Information. [file 41598_2021_2726_MOESM1_ESM.pdf]

Supplementary Materials

Supplementary Figure S1

Supplementary Figure S2

Supplementary Figure S3

Supplementary Table S1

Supplementary Table S2

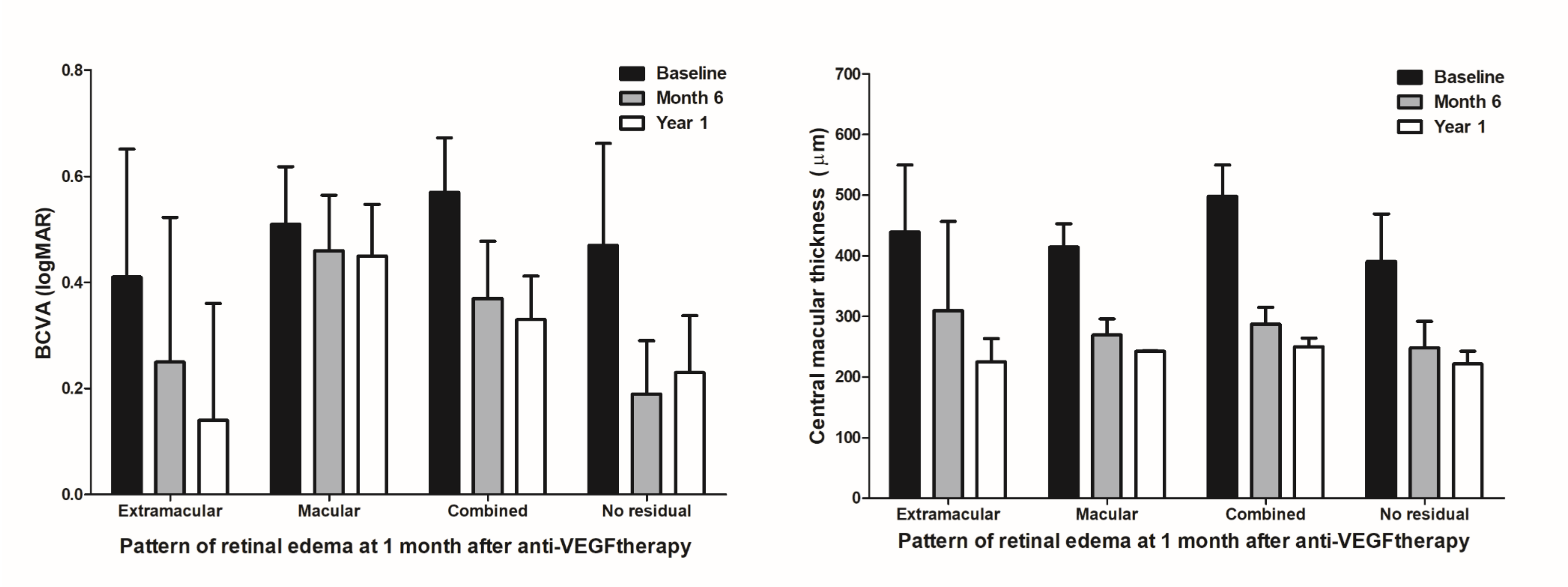

Supplementary Figure S1. The changes in best-corrected visual acuity (BCVA) and central macular thickness (CMT) over time during the 1-year follow-up period in subgroups separated based on the patterns of retinal edema at 1 month after anti-vascular endothelial growth factor therapy. Compared to baseline, BCVA improved remarkably at 6 months and 1 year, which corresponded to a decrease in CMT.

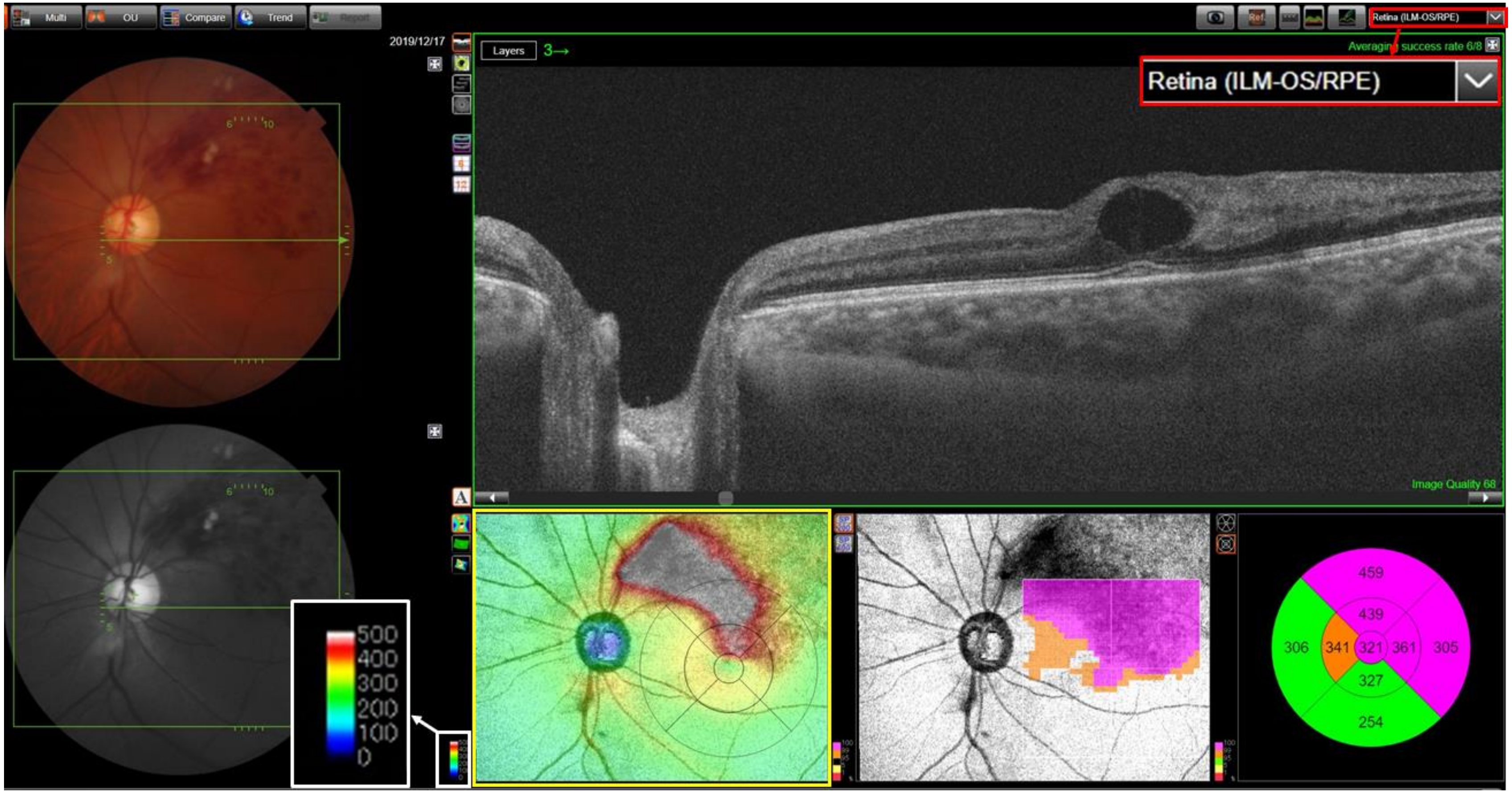

Supplementary Figure S2. Photograph of a wide-field retinal thickness map obtained by a 12 x 9 mm^2 macular volume scan of swept-source optical coherence tomography in an eye with branch retinal vein occlusion. A color-coded map (demarcated by the yellow box) is automatically generated after setting the retinal segmentation (red box) from the internal limiting membrane (ILM) to the outer segment/retinal pigment epithelium (OS/RPE). The map indicates areas with abnormal retinal thickening, as red or white (white box), and the location of edema, macular and/or extramacular, could be determined using an Early Treatment Diabetic Retinopathy Study grid over the macula.

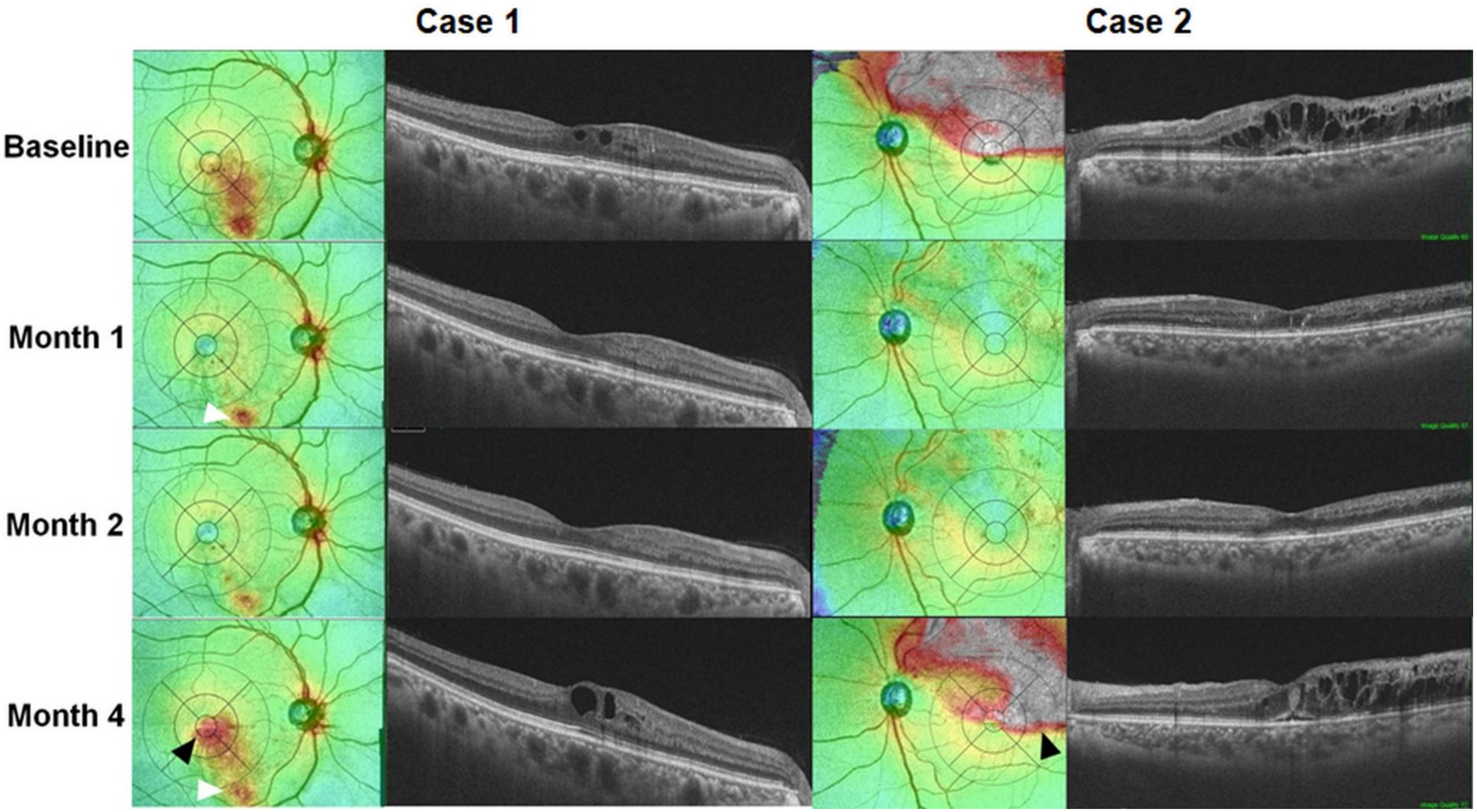

Supplementary Figure S3. Photographic examples demonstrating unusual evolution of macular edema (ME) recurrence. In contrast to the expansion and aggravation of residual edema noted 1 month after initial therapy in most cases with recurrence, these cases show ME recurrence independent from the residual edema (Case 1) or did not have any residual edema (Case 2). White arrowheads indicate residual edema, and black arrowheads denote recurred ME.

Supplementary Table S1. Comparison of baseline characteristics and treatment details of the included patients among groups separated according to baseline pattern of retinal edema (n = 87)

Characteristics	Macular edema only (n = 12)	Combined edema-macular dominant (n = 47)	Combined edema-extramacular dominant (n = 28)	P-value
Age, yr	66.6 ± 5.5	62.4 ± 10.5	64.3 ± 12.1	0.430
Sex, female (%)	7 (58.3%)	28 (59.6%)	17 (60.7%)	0.989
Location of vascular occlusion, Superotemporal (%)	7 (58.3%)	31 (66.0%)	11 (39.3%)	0.078
Subtype of BRVO, macular:major	11:1 (91.7:8.3)	11:36 (23.4:76.6)	15:13 (53.6:46.4)	**<0.001**
Initial BCVA, logMAR	0.47 ± 0.24	0.54 ± 0.30	0.55 ± 0.34	0.765
Follow-up period, months	33.6 ± 17.7	24.9 ± 20.2	30.2 ± 23.9	0.474
Central macular thickness, μm	412.3 ± 87.2	466.5 ± 155.5	450.9 ± 139	0.502
Anti-VEGF agents, bevacizumab:ranibizumab (%)	11:1 (91.7%:8.3%)	35:12 (74.5%:25.5%)	23:5 (82.1%:17.9%)	0.435
Eyes with 6-month recurrence (%)	8 (66.7%)	34 (72.3%)	21 (75%)	0.864
Number of recurrences during 1-year period	2.6 ± 1.6	2.3 ± 1.6	2.1 ± 1.5	0.673

Continuous values are denoted as mean ± standard deviation.

BCVA: Best-corrected visual acuity, VEGF: Vascular endothelial growth factor, logMAR: Logarithm of the minimum angle of resolution.

Supplementary Table S2. Comparison of baseline characteristics and treatment details of the included patients among groups separated according to the pattern of retinal edema 1 month after anti-vascular endothelial growth factor therapy (n = 87)

**Characteristics**	**No edema (n = 9)**	**Extramacular edema only (n = 6)**	**Macular edema only (n =30)**	**Combined edema (n = 42)**	**P-value**
Age, yr	60.7 ± 10.0	56.7 ± 6.9	64.8 ± 11	64.3 ± 10.5	0.276
Sex, female (%)	8 (88.9%)	3 (50.0%)	16 (53.3%)	25 (59.5%)	0.269
Location of vascular occlusion, Superotemporal (%)	3 (33.3%)	3 (50.0%)	15 (50.0%)	28 (66.7%)	0.227
Subtype of BRVO, macular:major (%)	5:4 (55.6:44.4%)	0:6 (0:100%)	20:10 (66.7:33.3%)	12:30 (28.6:71.4%)	**<0.001***
Initial BCVA, logMAR	0.47 ± 0.25	0.42 ± 0.23	0.51 ± 0.30	0.57 ± 0.33	0.548
Follow-up period, months	32.4 ± 29.2	19.0 ± 4.2	28.6 ± 18.5	26.9 ± 21.6	0.863
Central macular thickness, μm	390.4 ± 102.2	439.5 ± 104.9	414.3 ± 102.6	498.0 ± 166.5	**0.039***
Anti-VEGF agents, bevacizumab:ranibizumab (%)	8:1 (88.9:11.1%)	6:0 (100:0%)	24:6 (80:20%)	31:11 (73.8:26.2%)	0.415

Continuous values are denoted as mean ± standard deviation.

BCVA: Best-corrected visual acuity, VEGF: Vascular endothelial growth factor, logMAR: Logarithm of the minimum angle of resolution.

*P < 0.05
